# The latest progression of N6-methyladenosine (m^6^A) RNA modification in diabetes mellitus and diabetic complications

**DOI:** 10.3389/fendo.2025.1705600

**Published:** 2026-01-09

**Authors:** Guangyang Yin, Jiajing Hong, Guohui Shan, Cuizhu Xu, Bailin Li, Dongyu Yang

**Affiliations:** 1 Department of Chinese Medicine, Changchun Fukang Hospital, Changchun, China; 2 College of Acupuncture and Massage, Changchun University of Chinese Medicine, Changchun, China; 3 Department of Endocrinology, The Third Affiliated Hospital of Changchun University of Chinese Medicine, Changchun, China; 4 Medical Record Center, The Third Affiliated Hospital of Changchun University of Chinese Medicine, Changchun, China; 5 Medical Quality Monitoring Center, The Third Affiliated Hospital of Changchun University of Chinese Medicine, Changchun, China; 6 Center of Traditional Chinese Medicine, The Third Affiliated Hospital of Changchun University of Chinese Medicine, Changchun, China

**Keywords:** diabetes complications, diabetes mellitus, m^6^A, m^6^A regulators, methyladenosine

## Abstract

The increasing global prevalence of diabetes mellitus and its complications continues to encourage exploration of novel molecular mechanisms for their prevention and treatment. N6-methyladenosine (m^6^A) is a methylation modification that occurs at the N6 position of adenosine in most RNAs and represents the most prevalent internal modification of eukaryotic messenger RNA. This dynamic and reversible modification is involved in regulating nearly all aspects of RNA metabolism and therefore plays important roles in various diseases, including diabetes mellitus and its complications. The present review summarizes recent advances in understanding the functions of m^6^A modification, its regulators, and potential downstream targets in diabetes mellitus and diabetic complications. Notably, different—and sometimes opposite—expression patterns and regulatory roles of m^6^A regulators have been reported within the same disease or among diabetes-related disorders. The heterogeneity of patient tissues, cell lines, and experimental models used across studies highlights the need for further comprehensive evaluation of the roles of m6A modification in diabetes mellitus and its complications. This review provides a valuable reference for tracking recent research progress in the field of m^6^A modification in diabetes mellitus.

## Introduction

Diabetes mellitus (DM) is an endocrine disorder characterized by abnormally high blood glucose levels, and it is estimated to affect 693 million adults by 2045 (1). Diabetes can be diagnosed based on plasma glucose criteria, including a fasting plasma glucose (FPG) level of at least 126 mg/dL, a 2-h plasma glucose (2-h PG) level of at least 200 mg/dL during an oral glucose tolerance test, or a glycated hemoglobin A1C level of at least 6.5% (2). Diabetes can be classified into the following main categories: type 1 diabetes mellitus (T1DM), type 2 diabetes mellitus (T2DM), specific types of diabetes due to other causes, and gestational diabetes mellitus (GDM) (3). Diabetic complications mainly include macrovascular complications (e.g., cardiovascular disease) and microvascular complications (e.g., diabetic nephropathy, diabetic retinopathy, and diabetic neuropathy, etc.) (1, 4). Most patients with DM develop at least one complication; moreover, cardiovascular complications are the leading cause of morbidity and mortality among patients with DM (5).

Epigenetic mechanisms regulate gene activity and organismal development, and the epigenome plays a critical role in the development of DM and certain diabetic complications, including chromatin remodeling, DNA modifications, histone alterations, non-coding RNAs, and RNA modifications (6, 7). N(6)-methyladenosine (m^6^A) is the most prevalent, abundant, and conserved internal co-transcriptional modification in eukaryotic RNAs (8), which participates in nearly all aspects of RNA metabolism, including splicing, transcription, transport, stability, translation, and degradation (9, 10). Studies in recent years have shown that m^6^A RNA modification plays important roles in various physiological and pathological conditions (11), including DM and its complications (12–14). Although several reviews have summarized the role of m^6^A modification in diabetes, some focused only on T2DM (15, 16). In addition, other reviews were published earlier (17, 18), and did not incorporate the most recent studies on m^6^A modification in diabetes and diabetes-related complications.

**Figure 1:**
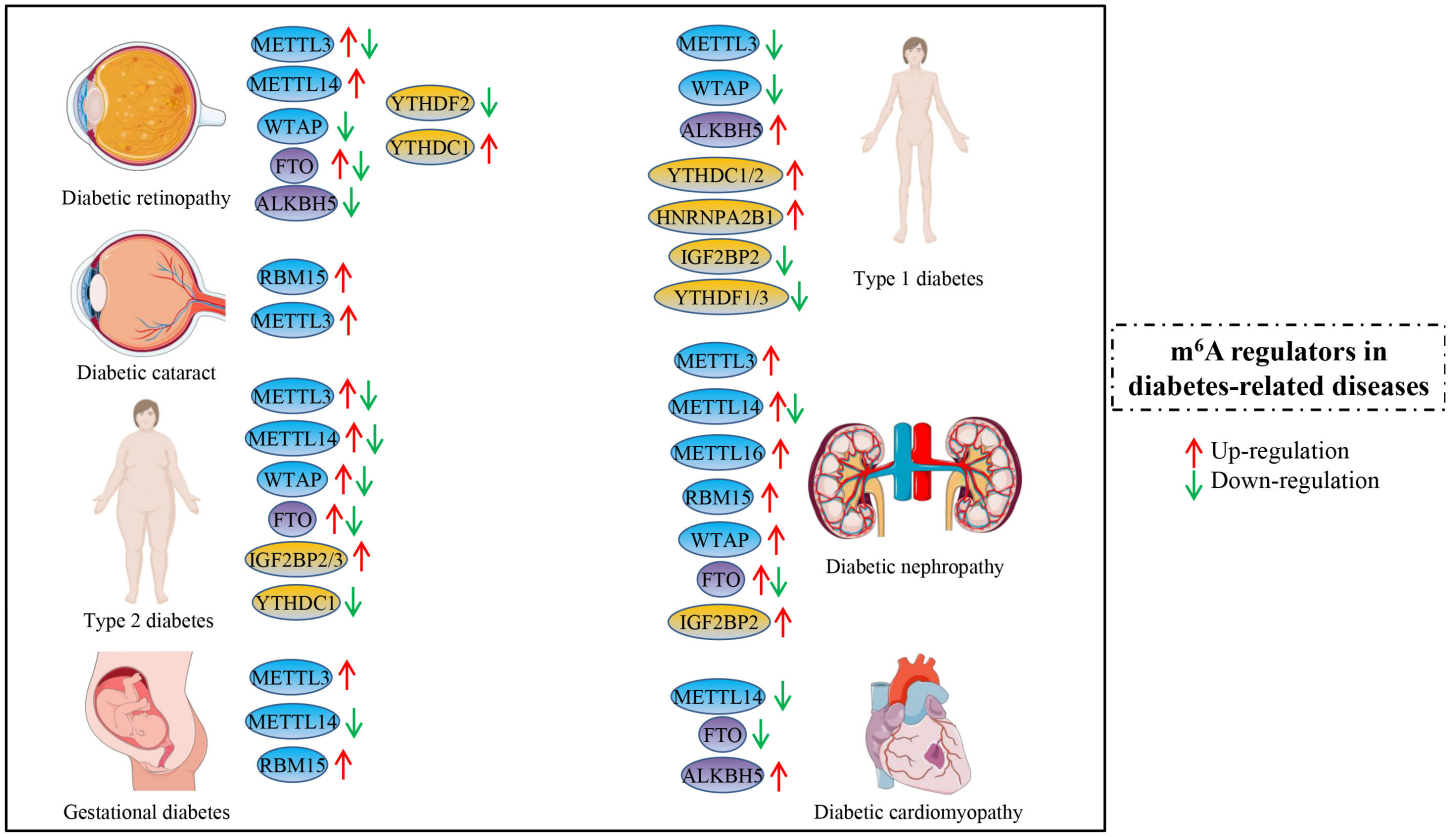
The expression and roles of some main m^6^A regulators (writers, erasers, and readers) in diabetes mellitus and diabetes-related complications, including diabetic retinopathy, diabetic cataract, type 2 diabetes, gestational diabetes, type 1 diabetes, diabetic nephropathy, and diabetic cardiomyopathy. The red arrow represents the up-regulation of specific m^6^A regulators, and the green arrow represents down-regulation. The co-occurrence of red and green arrows indicates potentially different roles of m^6^A regulators in one of the above diseases.

To comprehensively track m^6^A modification and emerging research trends in the diabetes field, this review summarizes recently published studies that focus on the associations between m^6^A RNA modification and DM or diabetes-related complications. More importantly, we present and discuss the diverse roles of m^6^A modification and its regulators across multiple diabetes-related diseases (**Figure 1**).

## m^6^A modification and its regulators

The effects of m^6^A modification on mRNA are mediated by a group of m^6^A writer complex components, erasers, and readers, which catalyze, remove, and recognize m^6^A modifications on RNA, thereby functioning in normal physiological processes and pathological conditions, respectively (19, 20). The m^6^A writers constitute a multicomponent methyltransferase complex, mainly including METTL3, METTL14, WTAP, KIAA1429, RBM15, and ZC3H13. Demethylases, also termed erasers, consist of FTO and ALKBH5. Reader proteins recognize m^6^A-modified RNA and bind to it and include nuclear m^6^A readers (YTHDC1, HNRNPA2B1, and HNRNPC) and cytoplasmic m^6^A readers (YTHDFs, IGF2BPs, and YTHDC2) (20, 21).

## The role of m^6^A modification and its regulators in diabetes and diabetes complications

### Type 1 diabetes mellitus

Type 1 diabetes mellitus (T1DM) is one of the main forms of DM and is an autoimmune disease characterized by insulin deficiency due to pancreatic β-cell loss, leading to hyperglycemia (22). The m^6^A regulators YTHDC1 and HNRNPA2B1 were significantly up-regulated in peripheral blood mononuclear cells (PBMCs) of patients with T1DM compared with healthy individuals; however, METTL3 and IGF2BP2 were down-regulated in T1DM (23). The methylation levels of differentially methylated transcripts were significantly associated with their expression levels (23). Interestingly, a recent study validated that METTL3 levels increased drastically in β-cells at T1DM onset but rapidly declined with disease progression. More importantly, METTL3 regulated several key innate immune mediators at the β-cell level during the onset of T1DM in humans (12). These two studies indicate that METTL3 expression in T1DM may eventually decrease but may experience a transient increase during the early stages of the disease, potentially due to cellular stress. Furthermore, differences in sample sources may account for the varying expression levels of METTL3 observed in T1DM. Another study revealed that three tag single-nucleotide polymorphisms (SNPs) located in introns of PRRC2A and YTHDC2 were associated with T1DM risk in a Chinese cohort of 1005 patients with T1DM and 1257 controls using a genome-wide association study (GWAS) microarray (24). The variant rs2260051 of PRRC2A was correlated with increased PRRC2A mRNA expression in PBMCs from patients with T1DM (24). These genomic and transcriptomic studies suggest that m^6^A modification and its regulators may play important roles in the pathogenesis of T1DM and could serve as potential targets for T1DM diagnosis or treatment.

Researchers constructed a T1DM mouse model using streptozotocin intraperitoneal injection, which exhibited marked cognitive dysfunction. Streptozotocin induction significantly up-regulated the protein expression of YTHDC2 and ALKBH5, while down-regulating YTHDF1, YTHDF3, and WTAP (25). Furthermore, overexpression of YTHDF1 in the hippocampus significantly improved streptozotocin-induced diabetic cognitive dysfunction (25). Based on m^6^A-related studies using various sample types from both humans and mice, it can be inferred that overall m^6^A modification levels may be decreased in T1DM (**Figure 1**).

### Type 2 diabetes mellitus

Type 2 diabetes mellitus (T2DM) is the most common form of diabetes, accounting for approximately 90% of patients with DM (5). T2DM is an increasingly prevalent multifactorial disease with both genetic and environmental risk factors, ultimately resulting in impaired glucose homeostasis (26). Shen et al. reported that global m^6^A levels were significantly lower in peripheral blood samples from patients with T2DM and diabetic rats compared with controls (**Table 1**). Notably, increased FTO expression may be responsible for the reduction in m^6^A levels in T2DM (27). Consistently, Yang et al. found decreased m6A content in blood samples from patients with T2DM, while mRNA expression levels of FTO, METTL3, METTL14, and WTAP were increased. FTO mRNA levels were positively correlated with fasting glucose levels (28). Coincidentally, Zhang et al. revealed that the m^6^A demethylase FTO was elevated in insulin-treated vascular smooth muscle cells (VSMCs) and in T2DM mice with intimal injury. Mechanistically, FTO knockdown increased SM22α expression, and the m^6^A reader IGF2BP2 enhanced SM22α mRNA stability (29). Ning et al. demonstrated that FTO expression was significantly up-regulated in trophoblasts from placentas of women with T2DM and that FTO-mediated m^6^A modification of SIK1 modulated placental cytotrophoblast syncytialization (30). Another study found that NR3C1 enhancement up-regulated FTO protein levels in β-cells and that the specific FTO inhibitor Dac51 effectively rescued NR3C1-induced β-cell failure, hyperglycemia, and glucose intolerance in diabetes models (31). However, another study reported opposing results regarding m^6^A and FTO levels in the blood of patients with T2DM. Although m^6^A modification levels were lower in obese and T2DM patients than in healthy controls, the demethylases FTO and ALKBH5 also showed reduced expression in the blood of both obese and T2DM patients (32). These findings suggest that decreased m^6^A methylation levels are consistently observed across different cohorts and sample types in T2DM, whereas the regulatory roles of FTO may be complex due to sample or cellular heterogeneity. Notably, recent research has shown that FTO preferentially produces a hemiaminal product and may function as a hydroxylase rather than a demethylase, unlike ALKBH5 (33). Therefore, further studies are required to clarify the demethylase-dependent and -independent roles of FTO in T2DM.

**Table 1:** The role of m6A modification and regulators in type 2 diabetes mellitus.

Main regulator	Expression in T2DM	Potential targets or pathways	Year	Reference
FTO	Up	/	2015	(27)
FTO	Up	FOXO1, G6PC, DGAT2	2019	(28)
FTO	Up	SM22α	2022	(29)
FTO	Up	SIK1	2024	(30)
FTO	Up	Atg12, Atg5, Atg16l2, Atg9a	2023	(31)
FTO and ALKBH5	Down	/	2022	(32)
Mettl14	Down	Insulin/IGF1-AKT-PDX1	2019	(13)
Mettl3/14	Down	MafA	2020	(36)
METTL3	Up	Fasn	2019	(37)
METTL14	Down	PDX1	2025	(38)
METTL3	Down	EGR1	2025	(39)
WTAP	Down	insulin secretion-related genes	2023	(40)
WTAP	Up	NT5DC3	2023	(41)
IMP2 and IGF2BP2	Down	PDX1	2021	(42)
IGF2BP3	Up	/	2023	(43)
YTHDC1	Down	insulin secretion-related genes	2023	(44)
YTHDC1	Down	SRSF3 and CPSF6	2023	(45)

Methyltransferases METTL3 and METTL14 have also been widely investigated in T2DM. m^6^A sequencing of human T2DM islets revealed that several hypomethylated transcripts were associated with cell-cycle progression, insulin secretion, and the insulin/IGF1–AKT–PDX1 signaling pathway. Impairment of insulin/IGF1–AKT–PDX1 signaling is a recognized mechanism in T2DM, in which PDX1 regulates β-cell identity and insulin secretion (34, 35). Importantly, β-cell-specific Mettl14 knockout mice displayed reduced m^6^A levels and recapitulated the islet phenotype observed in human T2DM, including early diabetes onset and increased mortality (13). This study highlighted a more prominent role for METTL14 in modulating the insulin/IGF1–AKT–PDX1 axis compared with METTL3 in T2DM islets. Reduced Mettl3/*Mettl14* expression was also observed in β-cells from diabetic db/db mice and patients with T2DM. Furthermore, mice with Mettl3/*Mettl14* deletion in Ngn3^+^ endocrine progenitors developed hyperglycemia and hypoinsulinemia within 2 weeks after birth (36). This study validated the roles of Mettl3/14 in neonatal β-cell development and functional maturation.

In contrast, another study reported that m^6^A levels and METTL3 expression were increased in liver tissues from patients with T2DM and mice fed a high-fat diet (HFD) for 16 weeks. Moreover, hepatocyte-specific *Mettl3* knockout in HFD-fed mice improved insulin sensitivity and reduced fatty acid synthesis (37). Luo et al. demonstrated that semaglutide alleviated β-cell dysfunction through METTL14 signaling in T2DM mice (38). Tao et al. showed that METTL3-mediated m^6^A modification of EGR1 mRNA promoted T2DM-associated vasculopathy (39). These studies suggested tissue-specific expression patterns and context-dependent functions of METTL3 and METTL14 in T2DM.

Several studies have reported the role of the methyltransferase WTAP in T2DM. WTAP was identified as being down-regulated in islet β-cells in T2DM, and islet β-cell–specific deletion of Wtap induced β-cell failure and diabetes. Moreover, Wtap-βKO mice exhibited severe hyperglycemia, which could be partially reversed by islet β-cell–specific overexpression of Mettl3 (40). The expression of NT5DC3 in blood samples could effectively distinguish patients with T2DM or T2DM-induced colon cancer from healthy volunteers, and its level was shown to be regulated by WTAP via m^6^A modification (41).

Other studies of T2DM focused on m^6^A readers. Deletion of IGF2BP2 in pancreatic β-cells led to reduced compensatory β-cell proliferation and impaired β-cell function, and IGF2BP2 directly bound to PDX1 mRNA and stimulated its translation in an m^6^A-dependent manner (42). Another study reported that IGF2BP2 and IGF2BP3 were up-regulated in islet samples from patients with T2DM, and a U-shaped association was observed between serum IGF2BP3 levels and the odds of T2DM in a Chinese adult population (43). YTHDC1 was found to be down-regulated in islet β-cells in T2DM, and β-cell–specific deletion of Ythdc1 resulted in β-cell failure and diabetes (44). Similarly, both m^6^A and YTHDC1 levels were reduced in patients with T2DM, and deletion of Ythdc1 in βKO mice caused glucose intolerance and diabetes due to decreased insulin secretion (45).

Public datasets have also been used to analyze the expression of m^6^A methylation regulators and their roles in gene regulation in T2DM (46, 47). The up-regulated expression of the m^6^A reader HNRNPC was shown to promote vascular endothelial dysfunction in T2DM by activating the PSEN1-mediated Notch pathway (46). In addition, co-expression gene analysis of m^6^A regulators identified several genes and pathways that have been reported to be strongly associated with T2DM (47).

## Diabetic retinopathy

Diabetic retinopathy (DR) is a common and specific microvascular complication of diabetes, and remains the leading cause of preventable blindness in working-aged people (48). METTL3 is widely studied m^6^A methyltransferase in DR (**Table 2**). In retinal pigment epithelium (RPE) cells, METTL3 expression was inhibited by high-glucose (HG) treatment in an *in vitro* model of DR, and up-regulation of METTL3 alleviated the cytotoxic effects of HG on RPE cells. Mechanistically, METTL3 rescued cell viability in HG-treated RPE cells by targeting the miR-25-3p/PTEN/Akt signaling pathway (49). Interestingly, the PTEN/Akt signaling has been widely reported as a downstream target of miRNAs or lncRNAs in the pathogenesis and improvement of DR (50–52). Similarly, METTL3 expression was significantly down-regulated in patients with DR, DR mouse models, and HG-induced human retinal microvascular endothelial cells (HRMECs). Further experiments demonstrated that METTL3 regulated endothelial–mesenchymal transition in DR via the SNHG7/KHSRP/MKL1 axis in an m^6^A modification (53). Notably, the lncRNA SNHG7 has also been reported to regulate endothelial–mesenchymal transition in DR through alternative mechanisms, including miRNA-mediated pathways (54).

**Table 2:** The role of m6A modification and regulators in diabetic retinopathy.

Main regulator	Expression in DR	Potential targets or pathways	Year	Reference
METTL3	Down	miR-25-3p/PTEN/Akt signaling	2020	(49)
METTL3	Down	SNHG7/MKL1 axis	2022	(53)
METTL3	Down	ANXA1	2022	(55)
METTL3	Down	PSAT1	2024	(56)
METTL3	Down	PIEZO1	2025	(57)
Mettl3/Wtap	Down	/	2023	(58)
METTL3	Up	PKC-η/FAT4/PDGFRA signaling	2022	(59)
METTL3	Up	OGRU	2025	(60)
YTHDF2	Down	LC3B	2022	(61)
YTHDF2	Down	ITGB1	2021	(62)
YTHDF2	Down	PARP1	2022	(63)
ALKBH5	Down	A20	2022	(64)
FTO	Up	CDK2	2024	(65)
FTO	Down	Nrf2/HO-1 signaling	2025	(66)
IGF2BP2	Up	HAGLR	2024	(67)
YTHDC1	Up	CDK6	2024	(68)
METTL14	Up	PHLPP2	2025	(69)

Blood–retinal barrier (BRB) breakdown contributes to multiple ocular diseases, including DR. One study found that endothelial CYP2J2 overexpression maintained BRB integrity and protected against retinal ganglion cell loss. CYP2J2 up-regulated METTL3 expression and promoted ANXA1 translation via m^6^A modification in endothelial cells, identifying a potential pathway for alleviating BRB impairment and DR progression (55). In addition, recent studies showed that METTL3 may mitigate HG-induced ARPE-19 cell damage partly by regulating the stability of PSAT1 mRNA (56), and that the protective effect of PIEZO1 silencing was mediated by METTL3/YTHDF2-dependent m^6^A modification in DR (57). Public dataset analyses also revealed that Mettl3 and Wtap were significantly down-regulated in DR samples, and that Mettl3 (AUC = 0.917) and Wtap (AUC = 0.972) could serve as potential diagnostic biomarkers for DR (58). However, some studies reported contrasting findings. m^6^A RNA methylation levels and METTL3 expression were significantly up-regulated in pericytes and mouse retinas following diabetic stress, and pericyte-specific *Mettl3* knockout inhibited diabetes-induced pericyte dysfunction and vascular complications *in vivo* (59). Fu et al. also reported that HG stimulation or diabetic stress increased both global m6A levels and METTL3 expression in experimental DR models (60).

The m^6^A reader protein YTHDF2 is the most extensively studied binding protein in DR. Circular RNA circFAT1 was significantly down-regulated in retinal proliferative fibrovascular membranes from patients with DR and in HG-induced RPE cells. circFAT1 promoted autophagy and inhibited HG-induced pyroptosis in RPE cells, potentially through regulation by the m^6^A reader YTHDF2 and their shared target LC3B (61). Up-regulation of KAT1 suppressed inflammation, neovascularization, and vascular leakage in retinal tissues of DR mouse models. YTHDF2 transcriptional activity was activated by KAT1 through histone acetylation of its promoter, leading to destabilization of target ITGB1 mRNA and attenuation of DR progression (62). PARP1 plays an important role in DR progression, and its inhibition prevented HG-induced inflammation, fibrosis, and angiogenesis in DR models *in vivo*. Moreover, YTHDF2 epigenetically suppressed m^6^A modification of PARP1 and regulated diabetes-induced PARP1 expression in DR (63).

A20 expression was reduced in glucose-treated retinal microglia and negatively regulated M1 polarization. The m^6^A demethylase ALKBH5 increased m^6^A modification of A20 in glucose-treated retinal microglia (64). Another demethylase, FTO, was shown to be driven by lactate-mediated histone lactylation in DR and was important for endothelial cell function by modulating CDK2 mRNA stability (65). Chen et al. demonstrated that FTO ameliorated HG-induced oxidative stress and cell apoptosis by activating the Nrf2/HO-1 signaling pathway in an m^6^A-dependent manner (66). These findings suggest that m^6^A demethylases may exert protective roles in DR.

Aberrantly up-regulated HAGLR was identified in serum samples from patients with DR and in HG-induced human RPE cells, and its knockdown attenuated HG-induced cytotoxic effects on apoptosis and pyroptosis. Furthermore, HAGLR expression was positively regulated by the m^6^A reader IGF2BP2 in an m^6^A-dependent manner (67). Zhou et al. reported that YTHDC1 overexpression impaired retinal vascular endothelial cell function by repressing CDK6 expression, suggesting a potential therapeutic target for DR (68). Chen et al. demonstrated that suppression of METTL14 improved HG-induced retinal ganglion cell damage and protected against DR by down-regulating PHLPP2 via m^6^A modification (69). In addition, recent research showed that urinary m6A concentration may serve as a potential diagnostic marker for DR, as measured by UPLC–MS/MS, providing a novel noninvasive diagnostic approach (70).

Collectively, these studies indicate that comprehensive evaluation of expression changes and functional roles of diverse m^6^A regulators across different sample types and experimental conditions is necessary in DR.

## Diabetic cataract

Diabetic cataract (DC) is a major cause of vision loss in patients with diabetes and occurs earlier and progresses more rapidly than cataracts in non-diabetic patients (71). There were minority reports about m^6^A regulation in DC. The m^6^A abundance was increased in anterior lens capsules from patients with DC compared with control subjects, and ferroptosis-related pathways were associated with m^6^A-modified mRNAs (72). In addition, m^6^A regulator RBM15 was verified to be up-regulated in samples from DC patients and SRA01/04 cells with HG medium treatment (72). Similarly, METTL3 expression and global m^6^A levels were increased in DC tissues and in HG-induced human lens epithelial cells. METTL3 knockdown promoted proliferation and suppressed HG-induced apoptosis in lens epithelial cells (73). Dong et al. reported that elevated METTL3 contributed to DC progression via m^6^A modification of SIRT1 mRNA, influencing cellular autophagy and senescence (74). RBM15 was also shown to promote HG-induced lens epithelial cell injury by inducing m^6^A modification of *PRNP* during DC (75). These studies indicated that m^6^A modification in lens epithelial cells plays important roles in DC progression.

## Gestational diabetes mellitus

Gestational diabetes mellitus (GDM) traditionally refers to abnormal glucose tolerance with onset or first recognition during pregnancy (76). The mRNA level of the methyltransferase METTL14 was significantly decreased in placental samples from patients with GDM compared with controls, and methylated RNA immunoprecipitation sequencing (MeRIP-seq) data revealed that most m^6^A peaks were reduced in 3’-untranslated regions (UTRs) and coding sequences (CDSs) near stop codons in placental samples from patients with GDM (77). Li et al. further demonstrated that METTL14 was down-regulated in placentas from patients with GDM and that METTL14-mediated silencing of the lncRNA XIST could alleviate GDM progression through m^6^A modification (78). Chen et al. constructed an *in vitro* GDM model using high-glucose (HG)–treated HTR8/SVneo cells. Consistently, METTL14 expression was decreased in the HG-induced GDM model and was subsequently up-regulated by fentanyl treatment. In addition, silencing of METTL14 reduced both m^6^A and mRNA levels in trophoblast cells, thereby neutralizing the effects of fentanyl on m^6^A and mRNA expression (79).

Interestingly, another study found that m^6^A levels were increased in cord blood samples from overweight or obese patients with GDM. Lipopolysaccharide (LPS) and glucose treatment induced increased m^6^A modification in human villous trophoblasts, whereas hesperidin reversed m^6^A levels in LPS- and HG-induced villous trophoblasts (80). Fang et al. validated that the global m^6^A methylation level of mRNA was significantly higher in the fetal liver of a GDM mouse model, and that up-regulated methyltransferase RBM15 suppressed insulin sensitivity and increased insulin resistance through m^6^A-mediated epigenetic regulation (81). Zhang et al. also reported increased m^6^A modification levels and METTL3 expression in placental tissues from patients with GDM, and identified circular RNA hsa_circ_0072380 as a potential downstream target (82). Collectively, these findings show contradictory results regarding m^6^A levels in GDM-related samples, highlighting the need for comprehensive evaluation of m^6^A modification and its regulators across different tissues, models, and clinical contexts in GDM.

Several bioinformatic studies have suggested that m^6^A modification is associated with immune infiltration and lncRNA regulation in GDM (83, 84). Differentially expressed m^6^A-related genes were significantly associated with monocyte infiltration in GDM, and seven genes could distinguish patients with GDM from normal controls, including CD81, CFH, FABP5, GBP1, GNG11, IL1RL1, and SLAMF6 (83). Another study identified an m^6^A-related module consisting of LINC00667, YTHDF3, MYC, and miR-33a-5p that could classify GDM with high accuracy (84). These findings provide novel insights and potential therapeutic targets for GDM.

## Diabetic nephropathy

Diabetic nephropathy (DN), one of the major microvascular complications of diabetes, is the most common cause of end-stage renal disease in developed countries (85). METTL3 and METTL14 are the most widely studied m^6^A regulators in DN (**Table 3**). METTL3 was reported to be up-regulated in podocytes from renal biopsies of patients with DN. Furthermore, METTL3 knockout significantly reduced inflammation and apoptosis in HG-stimulated podocytes and alleviated podocyte injury and albuminuria in streptozotocin-induced diabetic mice (14). Similarly, Wang et al. found that METTL3 expression and global m^6^A levels were significantly elevated in DN, and that METTL3 silencing alleviated DN-induced kidney injury through YTHDF2-mediated m^6^A modification of PINK1 (86). Liu et al. further demonstrated that METTL3 knockout enhanced the expression of circ-0000953, which alleviated podocyte injury and autophagy dysfunction in DN (87). Silencing of METTL3 inhibited proliferation, migration, and renal fibrosis in HG-induced HK-2 cells by regulating m^6^A modification of WISP1 (88). However, Tang et al. reported contrasting results, showing that METTL3 overexpression alleviated renal impairment and fibrosis *in vitro* and *in vivo* by promoting m^6^A modification of the target NDS2 (89). These findings suggest that the function of METTL3 in DN requires further comprehensive investigation.

**Table 3:** The role of m6A modification and regulators in diabetic nephropathy.

Main regulator	Expression in DN	Potential targets or pathways	Year	Reference
METTL3	Up	TIMP2	2022	(14)
METTL3	Up	PINK1	2023	(86)
METTL3	Up	circ-0000953	2024	(87)
METTL3	Up	WISP1	2024	(88)
METTL3	Down	NDS2	2022	(89)
METT14	Up	Sirt1	2021	(90)
WTAP and METTL14	Up	DKK3	2024	(91)
METT14	Up	α-klotho	2021	(92)
METT14	Up	TUG1	2023	(93)
METT14	Down	PI3K/Akt pathway, HDAC5	2021	(94)
METTL16 and RBM15	Up	AGE-RAGE pathway	2023	(98)
WTAP	Up	NLRP3	2022	(99)
WTAP	Up	ENO1	2024	(100)
WTAP	Up	TRIM22	2024	(101)
METTL7A	Up	CIDEC	2024	(102)
FTO	Down	SOCS1	2022	(103)
FTO	Down	NLRP3	2024	(104)
FTO	Down	Npas2/Hif-1alpha	2025	(105)
FTO	Down	ACC1	2025	(106)
FTO	Up	ENST00000436340	2023	(107)
FTO	Up	SAA2	2024	(108)
IGF2BP2	Up	circUBXN7	2023	(110)
IGF2BP3	Down	CAMK1	2024	(111)

In addition to METTL3, METTL14 has been widely reported to be up-regulated in the kidneys of mice and patients with DN (90, 91). Podocyte-specific deletion of METTL14 improved glomerular function and alleviated podocyte injury in mice. *In vitro*, METTL14 knockdown in podocytes facilitated autophagy and reduced apoptosis and inflammation under adriamycin treatment (90). Li et al. reported that METTL14 was elevated in kidneys from patients with DN and in HG-induced human renal glomerular endothelial cells, and that METTL14 aggravated renal injury and inflammation in db/db mice by regulating α-klotho expression in an m^6^A-dependent manner (92). Zheng et al. further demonstrated that METTL14 knockdown protected against streptozotocin-induced renal lesions and renal fibrosis in DN mice by regulating the stability and expression of *TUG1* through m^6^A modification (93).

Conversely, other studies reported that METTL14 overexpression in HG-treated HK-2 cells led to inactivation of the PI3K/Akt signaling pathway and down-regulation of HDAC5, which are closely associated with epithelial–mesenchymal transition (EMT) in renal tubular cells during DN progression (94) EMT of renal tubular epithelial cells is a key driver of renal fibrosis in DN, and the PI3K/Akt pathway is widely involved in this process (95–97).

Other methyltransferases, including METTL16 and RBM15, were also found to be up-regulated in DN models. RBM15 silencing restored cell proliferation, reduced inflammation, and inhibited pyroptosis in HG-induced HK-2 cells (98). Coincidentally, WTAP expression was elevated in patients with DN and in HG-treated HK-2 cells, and WTAP knockdown attenuated HG-induced pyroptosis and NLRP3-related pro-inflammatory cytokine production in HK-2 cells and db/db mice (99).

Recently, multiple studies have reported that elevated WTAP expression may promote the progression and related pathological functions of DN by regulating different downstream targets via m^6^A modification (91, 100, 101). Jin et al. reported that adipose-derived stem cell–derived exosomal miR-204 could alleviate mitochondrial dysfunction in DN by inhibiting METTL7A-mediated m^6^A methylation of *CIDEC* (102). Although these studies revealed distinct regulatory mechanisms involving m^6^A methyltransferases, most methyltransferases were consistently up-regulated in DN or DN-related cellular models, leading to increased m^6^A modification of various downstream targets.

Fat mass and obesity-associated protein (FTO) is a widely studied m6A demethylase. Sun et al. revealed that FTO expression was significantly decreased in patients with DN, and that FTO overexpression markedly alleviated renal inflammation by increasing SOCS1 protein levels via m^6^A modification in db/db mice (103). FTO overexpression also alleviated kidney injury and suppressed DN-induced pyroptosis by regulating m^6^A modification of NLRP3 (104). Another two recent studies further validated the protective roles of FTO in DN (105, 106). However, Hu et al. demonstrated that FTO-mediated m^6^A modification induced up-regulation of the novel lncRNA ENST00000436340, which promoted podocyte injury in DN (107). Similarly, Lang et al. reported that FTO-mediated m^6^A modification of SAA2 mRNA promoted podocyte injury and inflammation in DN by activating the NF-κB signaling pathway (108). Importantly, NF-κB signaling has emerged as a promising therapeutic target for mitigating DN-associated inflammation and complications (109). Collectively, these studies indicate context-dependent and target-specific roles of FTO-mediated demethylation in DN.

m^6^A readers have been less extensively studied as primary regulators in DN. IGF2BP2 was shown to form an RNA–protein complex with SP1 and circUBXN7, which promoted macrophage infiltration and renal fibrosis in DN (110). In contrast, IGF2BP3-stabilized CAMK1 alleviated DN by regulating mitochondrial dynamics of renal tubule (111). Interestingly, urinary m^6^A levels were decreased in patients with DN and could distinguish individuals with DN from those with normal glucose tolerance (112). In addition, public datasets have been used to analyze m^6^A-modified gene expression in DN (113, 114). Both studies reported that m^6^A-related mRNAs or lncRNAs were correlated with M1 macrophage infiltration in DN; however, these findings require further experimental validation.

## Diabetic cardiomyopathy

Diabetic cardiomyopathy (DCM) is a primary myocardial injury induced by diabetes, which has been defined as the presence of abnormalities in myocardial structure and function that occur in the absence of well-established cardiovascular risk factors (115). A recent study validated that the global m^6^A level of heart samples was relatively higher in a DCM model with leptin receptor deficiency than in normal mice, and the MeRIP-seq results of DCM and normal hearts revealed that differentially m^6^A-modified genes were related to cardiac fibrosis, myocardial hypertrophy, and myocardial energy metabolism in DCM (116). In addition, up-regulated FTO could improve cardiac function by reducing myocardial fibrosis and myocyte hypertrophy in this DCM model (116). However, another study found that METTL14 was down-regulated in cardiomyocytes and heart tissues of a DCM rat model with high-glucose (HG) treatment, and its overexpression suppressed pyroptosis and DCM by down-regulating the lncRNA TINCR with the participation of the m^6^A reader protein YTHDF2 (117). Li et al. validated that recruitment of METTL14 to Casq2 mRNA could increase its stability via m^6^A modification, and Casq2 might act as a potential target of the lncRNA Trdn-as, which was remarkably up-regulated in the hearts of DCM mice and cardiomyocytes treated with HG (118).

Meng et al. found that ALKBH5 reduced the mRNA stability of SPOP by decreasing m^6^A modification, and overexpression of SPOP could improve ferroptosis and DCM-induced myocardial dysfunction (119). Similarly, ALKBH5 was found to reduce m^6^A methylation levels of Kat2a mRNA, and its inhibition could effectively ameliorate HG-induced cardiomyocyte injury by suppressing ferroptosis (120). The down-regulated m^6^A level of cardiac tissue was further validated in HG-induced DCM mice, and ALKBH5 was up-regulated in cardiomyocytes of DCM mice and post-transcriptionally activated FOXO3 via m^6^A demethylation (121). It seemed that the levels of both m^6^A-related regulators and m^6^A were opposite in different DCM models; therefore, the regulatory role of m^6^A in diabetic cardiomyopathy needs to be comprehensively investigated.

## Conclusion

Globally, the number of people with DM has quadrupled in the past three decades, and DM is the ninth major cause of death worldwide (5). However, the detailed mechanisms underlying the pathogenesis and progression of DM and its complications remain unclear. m^6^A RNA methylation provides new insights into the molecular mechanisms of DM and may help identify novel targets for the treatment of DM and DM-related diseases.

In this review, we summarized the different roles of m^6^A modification and its regulators in DM and its complications. These studies demonstrated the broad involvement of m^6^A modification in diabetic diseases. Moreover, m^6^A modification and most regulators showed similar expression signatures (up-regulation or down-regulation) or roles in specific diseases across different studies. However, some studies revealed that the same regulator might have different, or even opposite, roles within the same disease, indicating significant heterogeneity among samples, cell lines, and animal models. This suggests that systematic investigation under specific conditions using larger sample sizes, more cell types, and more sophisticated animal models is required. In addition, future studies should analyze the tissue-specific expression of m^6^A regulators in diabetic diseases, the RNA diversity of m^6^A modification, and the cell-type specificity of m^6^A methylation and its regulators at the single-cell level in specific tissues.
